# Assessment of the functional severity of coronary lesions from optical coherence tomography based on ensembled learning

**DOI:** 10.1186/s12938-023-01192-x

**Published:** 2023-12-16

**Authors:** Irina-Andra Tache, Cosmin-Andrei Hatfaludi, Andrei Puiu, Lucian Mihai Itu, Nicoleta-Monica Popa-Fotea, Lucian Calmac, Alexandru Scafa-Udriste

**Affiliations:** 1 Department of Automatic Control and Systems Engineering, University Politehnica of Bucharest, Bucharest, Romania; 2 Siemens Advanta SRL, 15 Noiembrie Bvd, 500097 Brasov, Romania; 3 Department of Automation and Information Technology, Transilvania University of Brasov, Mihai Viteazu Nr. 5, 5000174 Brasov, Romania; 4 Department of Cardiology, Emergency Clinical Hospital, 8 Calea Floreasca, 014461 Bucharest, Romania; 5 Department Cardio-Thoracic, University of Medicine and Pharmacy “Carol Davila”, 8 Eroii Sanitari, 050474 Bucharest, Romania; 6 Romanian Academy of Scientists, Bucharest, Romania

**Keywords:** Fractional flow reserve, Optical coherence tomography, Stenosis, Supervised learning, Ensemble model

## Abstract

**Background:**

Atherosclerosis is one of the most frequent cardiovascular diseases. The dilemma faced by physicians is whether to treat or postpone the revascularization of lesions that fall within the intermediate range given by an invasive fractional flow reserve (FFR) measurement. The paper presents a monocentric study for lesions significance assessment that can potentially cause ischemia on the large coronary arteries.

**Methods:**

A new dataset is acquired, comprising the optical coherence tomography (OCT) images, clinical parameters, echocardiography and FFR measurements collected from 80 patients with 102 lesions, with stable multivessel coronary artery disease. Having the ground truth given by the invasive FFR measurement, the dataset is challenging because almost 40% of the lesions are in the gray zone, having an FFR value between 0.75 and 0.85. Twenty-six features are extracted from OCT images, clinical characteristics, and echocardiography and the most relevant are identified by examining the models’ accuracy. An ensembled learning is performed for solving the binary classification problem of lesion significance considering the leave-one-out cross-validation approach.

**Results:**

Ensemble models are designed from the multi-features voting from 5 features models by prediction aggregation with a maximum accuracy of 81.37% and a maximum area under the curve score (AUC) of 0.856.

**Conclusions:**

The proposed explainable supervised learning-based lesion classification is a new method that can be improved by training with a larger multicenter dataset for further designing a tool for guiding the decision making of the clinician for the cases outside the gray zone and for the other situation extra clinical information about the lesion is needed.

## Background

Cardiovascular diseases, especially coronary artery disease (CAD), currently affect a major part of the population, along with the presence of arterial hypertension (AHT) [1]. The diagnosis and treatment of these patients is costly in terms of human and material resources. Many efforts are directed toward improving clinical management by reducing the diagnosis time interval, creating multidisciplinary teams, and optimizing the material costs.

In this context, medical imaging is helpful, and because of that, it is a continually growing domain that unifies the efforts of physicians, scientists, and engineers. Its impact on daily clinical management is high [2], especially in cardiovascular diseases and cancer, the two leading causes of death worldwide. This was empowered by the growth of computational power and data storage, which enable more accurate image classification, object detection, and image enhancement by means of image processing [3].

One of the most important cardiovascular diseases is atherosclerosis, which consists of a narrowing of the blood vessel lumen, mainly due to fat deposits. It may affect the major coronary arteries, causing myocardial ischemia at different degrees of exertion [4].

Optical coherence tomography (OCT) is acquired during invasive coronary X-ray angiography (XA), and it provides high-resolution images of the proximal coronary arteries with accurate quantification of the lumen and the structure of the vessel walls [5]. It may be used in the planning of interventional clinical procedures by selecting the diameter and length of the stents, the need for additional lesion preparation for highly calcified lesions and in the evaluation of stent expansion, apposition, and the presence of dissections. Direct measures of coronary stenoses, such as minimal luminal area (MLA) or minimal luminal diameter (MLD), are generally used for this aim [6, 7].

At the time of the invasive clinical procedure, the functional significance of a stenosis is optimally classified based on the evaluation of the fractional flow reserve (FFR), which is measured as the ratio between the pressure distal to the stenosis and the aortic pressure. In the medical literature and practice, the optimal cutoff value is 0.8 [8, 9]. A value less than 0.8 indicates functionally significant stenosis, requiring a revascularization procedure (either surgical or interventional) in addition to medical treatment.

Disadvantages of the invasive FFR are:

- Invasive procedure which involves inserting a catheter into the arteries. This procedure carries inherent risks such as bleeding, infection, and artery damage.
- Patient discomfort because it can cause pain and anxiety for patients. It often involves the use of a contrast agent, which may have side effects, and typically requires patients to remain still for an extended period.
- Additional costs ranging from 500 to 2000 euros are needed when compared with non-invasive methods due to the equipment and expertise required for the catheterization procedure.
- Additional time both for the patient in terms of preparation and recovery.

The gray zone with high uncertainty regarding the lesion significance is generally considered in the interval of 0.75 ≤ FFR ≤ 0.85 for which the physician will need extra information including the invasive FFR measurement.

Generally, the lesions situated in the gray zone have the medical recommendation for revascularization. Still, the physician must consider the clinical procedure risks and the overall clinical state of the patient (age, obesity, etc.) for deciding the treatment.

Virtual FFR (vFFR) can be estimated via machine learning (ML) algorithms from XA [10], OCT [11], intravascular ultrasound (IVUS) [12] and combinations of OCT and IVUS imaging [13] based on the computational fluid dynamics (CFD). All these approaches rely on features that characterize the vascular geometry, specifically the arterial lumen, and on clinical parameters.

Virtual FFR-based XA has the potential to alter decision making and it can increase the operators’ confidence in their decision [14].

Intracoronary OCT and FFR were acquired for the left anterior descending artery lesions in [11] for 125 patients with an accuracy of the OCT-based machine learning algorithm of 95.2%.

A total of 41 coronary stenoses in 30 patients were assessed consecutively in the paper [15] by quantitative coronary angiography (QCA), FFR, and intracoronary OCT. The study revealed that the diagnostic capability of MLA and MLD in identifying significant stenoses was moderate, with an area under the curve (AUC) of 0.80 for MLA and 0.76 for MLD. The optimal cutoff of OCT-measured MLA to identify stenoses with FFR ≤ 0.80 was 1.62 mm^2^.

FFR estimation from intracoronary OCT imaging based on CFD modeling, also known as OCT-based optical flow ratio (OFR), was addressed in [16], and a prototype software package (OctPlus) was built. Bifurcation fractal laws were applied to correct the step-down phenomenon lumen size for 125 vessels from 118 patients, with an average FFR of 0.80 ± 0.09. The overall vessel-level diagnostic accuracy was 90%, with a sensitivity and specificity of 87% and 92%, respectively.

Another study aimed to evaluate the diagnostic performance of the OFR [17] to compare it with the angiography-based quantitative flow ratio (QFR), using wire-based FFR as the gold standard in 212 vessels from 181 patients. The average FFR was 0.82 ± 0.10, and 40.1% of vessels had an FFR ≤ 0.80. The diagnostic accuracy, sensitivity, and specificity of OFR to identify FFR ≤ 0.80 were 92%, 86% and 95%, respectively.

An angiography-based machine learning (ML) algorithm was developed in [18] to classify lesions based on FFR cutoff value with an overall accuracy of 82% and AUC of 0.87.

The goal of the present study is to solve the binary classification problem for predicting the intermediate coronary lesions significance based on the patient’s medical characteristics and the features extracted from the lumen radii from intracoronary OCT.

## Results

### Population characteristics

Baseline patient and lesion characteristics are summarized in Tables 1 and 2: 80 patients with 102 intermediate coronary lesions are included in this study, where 57 are located on the left anterior descending coronary artery (LAD) with a mean FFR of 0.76, 20 on the left circumflex artery (LCX) with a mean FFR of 0.86, and 25 on the right coronary artery (RCA) with a mean FFR of 0.83.

**Table 1:** Baseline patient characteristics and risk factors (*n* = 80)

Male	66 (82%)	Hypertension	60 (75%)
Female	14 (18%)	Hypercholesterolemia	62 (77.5%)
Age (years)	60.5 ± 11.2 years	Smoking history	42 (52.5%)
Race	All Caucasian	Family history of CAD	3 (2.9%)
BMI	27.7 ± 2.5 kg/m^2^	Previous myocardial infarction	46 (45%)
Diabetes	27 (33.75%)	Ejection fraction	48.28 ± 6.31%
Previous Angina	64 (80%)

**Table 2:** Lesion characteristics (*n* = 102)

Fractional flow reserve		Index artery	
Mean ± SD	0.80 ± 0.08	LAD	57
Median (IQR)	0.83 (0.75–0.86)	LCX	20
FFR ≤ 0.80	48	RCA	25
FFR < 0.75	25		
0.75 ≤ FFR ≤ 0.85	47		
FFR > 0.85	30		

**Fig. 1:**
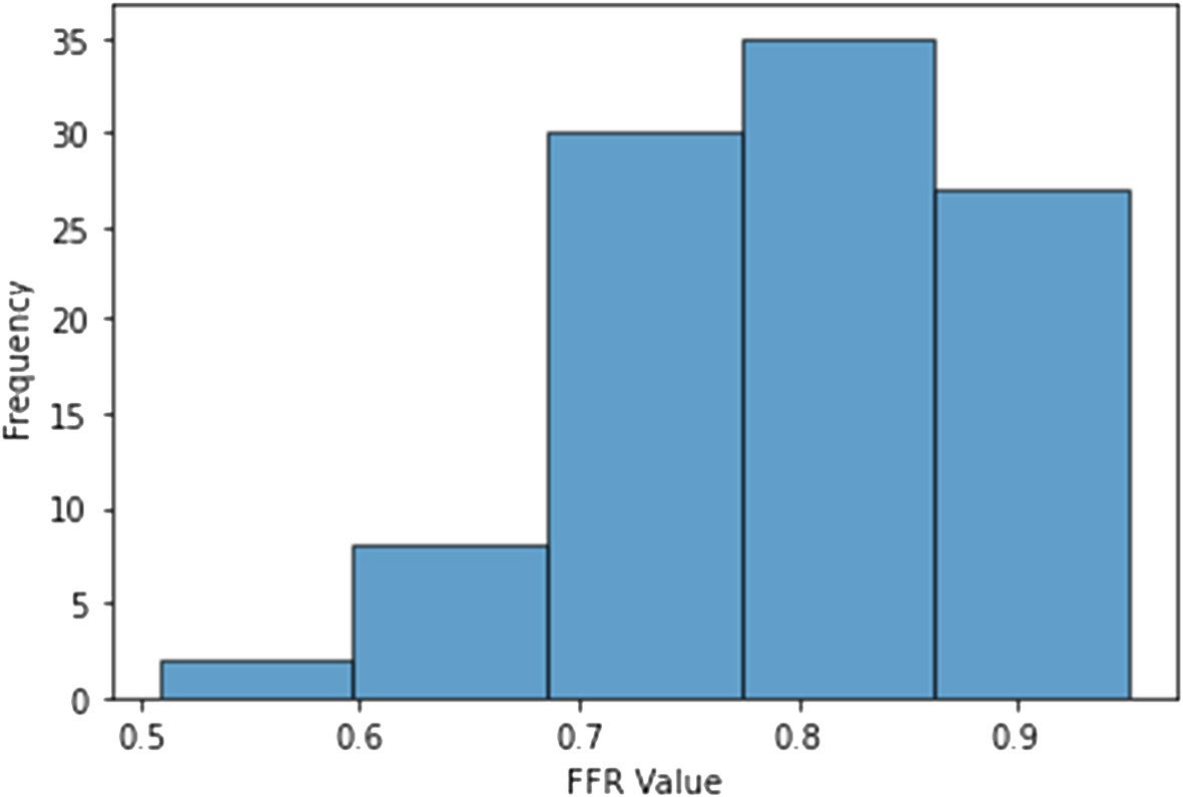
Histogram of the FFR values

The histogram of the corrected FFR values is presented in Fig. 1 to reveal the data concentration around the gray zone. This is justified by the fact that the physicians generally perform invasive FFR measurements when there is an uncertainty in the decision-making of revascularization.

### Lesion severity classification performance

The tenfold cross-validation strategy was applied for each univariate model with the same random seed variation. Each one of the features was analyzed with the following algorithms: SVM with different kernels, decision tree, k-nearest neighbors, random forest with different numbers of trees [19], AdaBoost [20] and naive Bayes [21]. From a total of 12 algorithms, only the one with the highest accuracy was selected in Table 3.

**Table 3:** Accuracies of the tenfold individual feature models

No	Feature input	ML algorithm	Mean Acc	Standard deviation	Max	Min
1	Minimal lumen diameter	Scale SVM	63.43	6.46	71.57	53.92
2	Proximal radius rapport	Poly SVM	64.31	5.14	71.57	53.92
3	Radius rapport	Poly SVM	65.59	4.39	71.57	54.9
4	Percentage diameter reduction	Poly SVM	66.18	1.4	68.63	63.73
5	Mean radius	Decision Tree	62.75	6.75	71.57	53.92
6	Minimum lumen radius	Naive Bayes	60.59	6.06	71.57	53.92
7	Weight	Naive Bayes	64.51	4.27	69.61	53.92
8	Mean lumen radius for stenosis region	Scale SVM	67.35	2.01	71.57	64.71
9	Mean radius per length	Scale SVM	61.76	6	71.57	53.92
10	Maximum radius rapport	K-nearest neighbors	64.31	5.79	71.57	53.92
11	Stenosis lesion length	Poly SVM	66.27	1.53	68.63	63.73
12	Hematocrit level	Decision Tree	62.25	6.59	71.57	53.92
13	Interventricular septum	Scale SVM	64.61	4.54	71.57	53.92
14	Maximum lumen radius	AdaBoost	65.39	2.98	69.61	58.82
15	Smoking	Scale SVM	55.5	4.8	59.8	47.52
16	Dyslipidemia	Naive Bayes	54.71	6.51	60.54	43.88
17	AHT	Scale SVM	53.53	3.72	56.86	47.34
18	Diastolic pattern	Naive Bayes	52.88	4.44	56.86	45.5
19	Age	Linear SVM	51.17	3.64	54.43	45.11
20	Diabetes	Random Forest 60	50.33	4.01	53.92	43.66
21	Distal area	Decision Tree	50.53	3.79	53.92	44.22
22	Echo EF	Poly SVM	49.59	4.83	53.92	41.56
23	Hb	Linear SVM	48.72	5.8	53.92	39.06
24	Hight	Random Forest 60	51.43	2.78	53.92	46.8
25	Proximal area	Linear SVM	50.52	3.8	53.92	44.2
26	Sex	Linear SVM	48.59	5.94	53.92	38.7

**Fig. 2:**
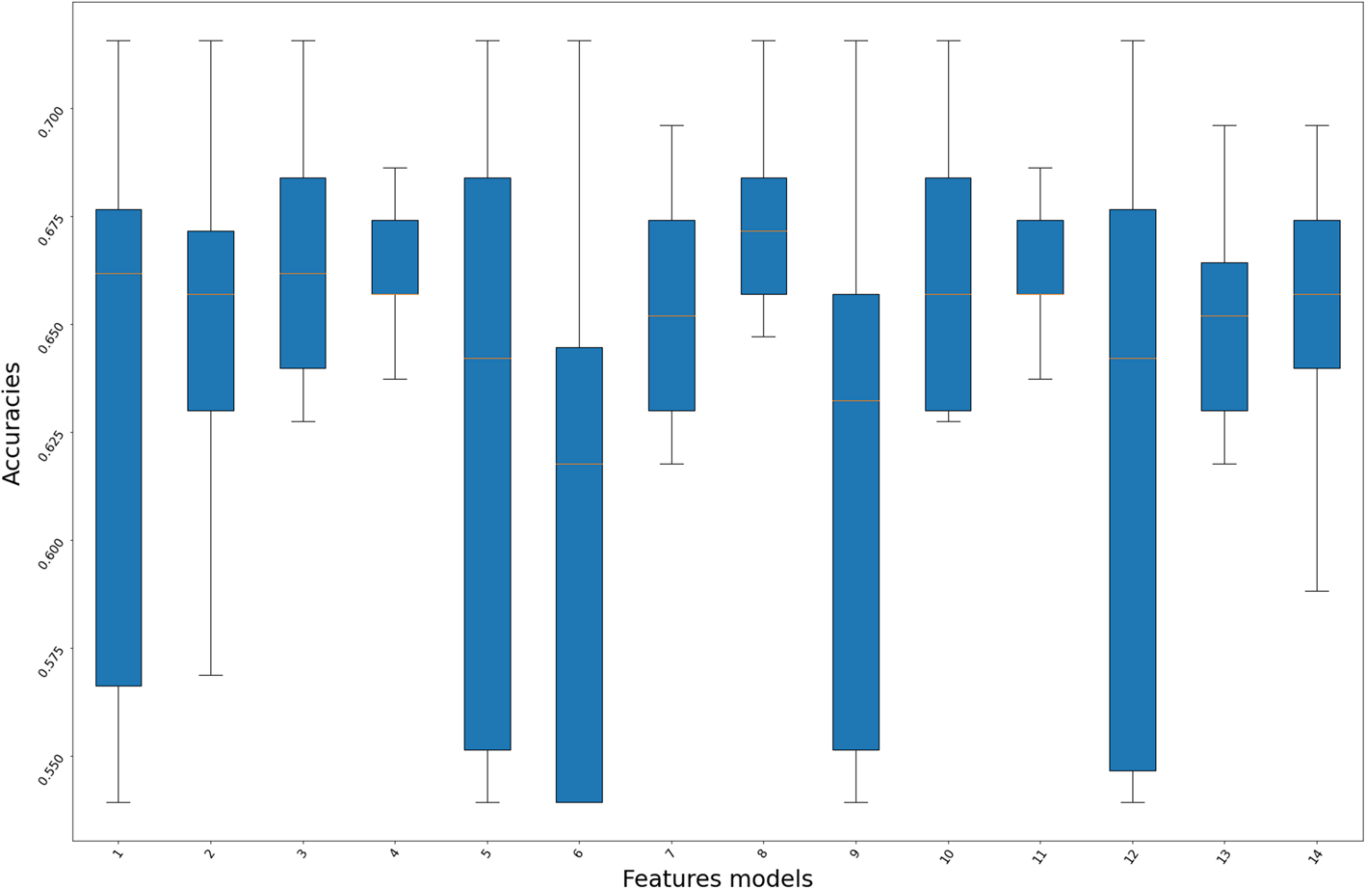
Box plot on the accuracies of the top 14 feature models

The models with a maximum accuracy of 60% (the top 14 feature models) were further analyzed through a box plot [22], as shown in Fig. 2. Two features have strong correlation, and radius rapport was eliminated from the study.

**Fig. 3:**
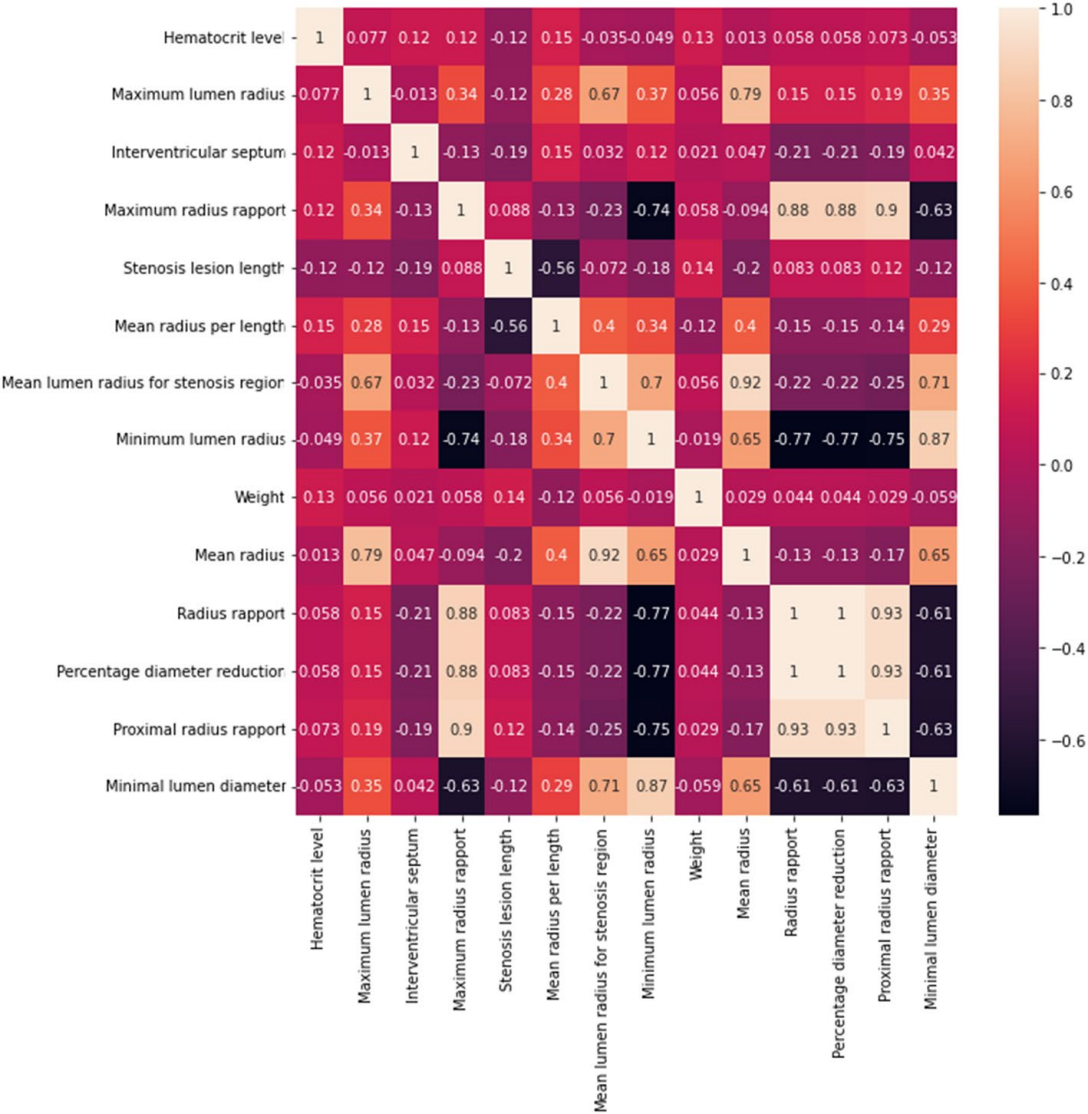
Correlation matrix for top 14 features

For feature analysis, the correlation matrix was computed and shown in Fig. 3, from where it can be observed that two features, radius rapport and percentage diameter reduction have perfect correlation, hence, the last one was eliminated from the feature map.

Some OCT features, for example, percentage diameter reduction and proximal radius rapport, mean radius, and mean lumen radius for stenosis region, have good positive or negative correlations due to their linear computation formulas (see Table 9). Still, in practice, if one of the multicollinear feature is removed, the ensemble model performance is decreasing, suggesting that their combination in voting is valuable.

In the simple and weighted voting approach, the accuracy is improved after running the ROC analysis and the models’ aggregation for the top 13 models. The performances of the ensembled model are displayed in Table 4 along with their corresponding AUC scores in Table 5.

**Table 4:** The ensemble models accuracies with a confidence interval of 95% obtained after ROC analysis using weighted voting

No. of feat	Class probabilities for all features using the mean ROC cutoff values (M1) (%)	Class probabilities using the corresponding ROC cutoff for each feature (M2) (%)	Crisp labels for all features using the mean ROC cutoff value (M3) (%)
1	75.49 (66.32,83.0)	75.49 (66.32,83.0)	73.53 (64.23,81.0)
2	76.47 (67.37,84.0)	72.55 (63.19,80.0)	73.53 (64.23,81.0)
3	78.43 (69.5,85.0)	73.53 (64.23,81.0)	79.41 (70.57,86.0)
4	77.45 (68.43,84.0)	71.57 (62.16,79.0)	74.51 (65.27,82.0)
5	75.49 (66.32,83.0)	68.63 (59.09,77.0)	81.37 (72.73,88.0)
6	73.53 (64.23,81.0)	69.61 (60.1,78.0)	79.41 (70.57,86.0)
7	75.49 (66.32,83.0)	71.57 (62.16,79.0)	80.39 (71.65,87.0)
8	73.53 (64.23,81.0)	70.59 (61.13,79.0)	80.39 (71.65,87.0)
9	75.49 (66.32,83.0)	72.55 (63.19,80.0)	80.39 (71.65,87.0)
10	76.47 (67.37,84.0)	69.61 (60.1,78.0)	80.39 (71.65,87.0)
11	75.49 (66.32,83.0)	69.61 (60.1,78.0)	80.39 (71.65,87.0)
12	77.45 (68.43,84.0)	70.59 (61.13,79.0)	79.41 (70.57,86.0)
13	77.45 (68.43,84.0)	71.57 (62.16,79.0)	80.39 (71.65,87.0)

**Table 5:** The AUC scores with their lower and upper limits using weighted voting

No. of feat	AUC using crisp labels	AUC using class probability
1	0.811 (0.725,0.897)	0.81 (0.724,0.896)
2	0.826 (0.743,0.909)	0.796 (0.707,0.885)
3	0.814 (0.729,0.9)	0.826 (0.743,0.909)
4	0.824 (0.74,0.907)	0.786 (0.695,0.877)
5	0.828 (0.745,0.91)	0.738 (0.64,0.836)
6	0.852 (0.775,0.929)	0.807 (0.64,0.894)
7	0.815 (0.73,0.901)	0.822 (0.739,0.906)
8	0.856 (0.78,0.933)	0.78 (0.689,0.872)
9	0.832 (0.75,0.914)	0.784 (0.693,0.875)
10	0.815 (0.73,0.901)	0.825 (0.742,0.908)
11	0.81 (0.724,0.897)	0.783 (0.691,0.874)
12	0.822 (0.738,0.906)	0.786 (0.696,0.877)
13	0.856 (0.78,0.932)	0.815 (0.73,0.901)

The model aggregation of the crisp labels of all features using the mean ROC cutoff value (M3 method) for the voting combination of 7, 8, 9, 10, 11, 13 features models led to the same evaluation metrics with an accuracy of 80.39%.

**Fig. 4:**
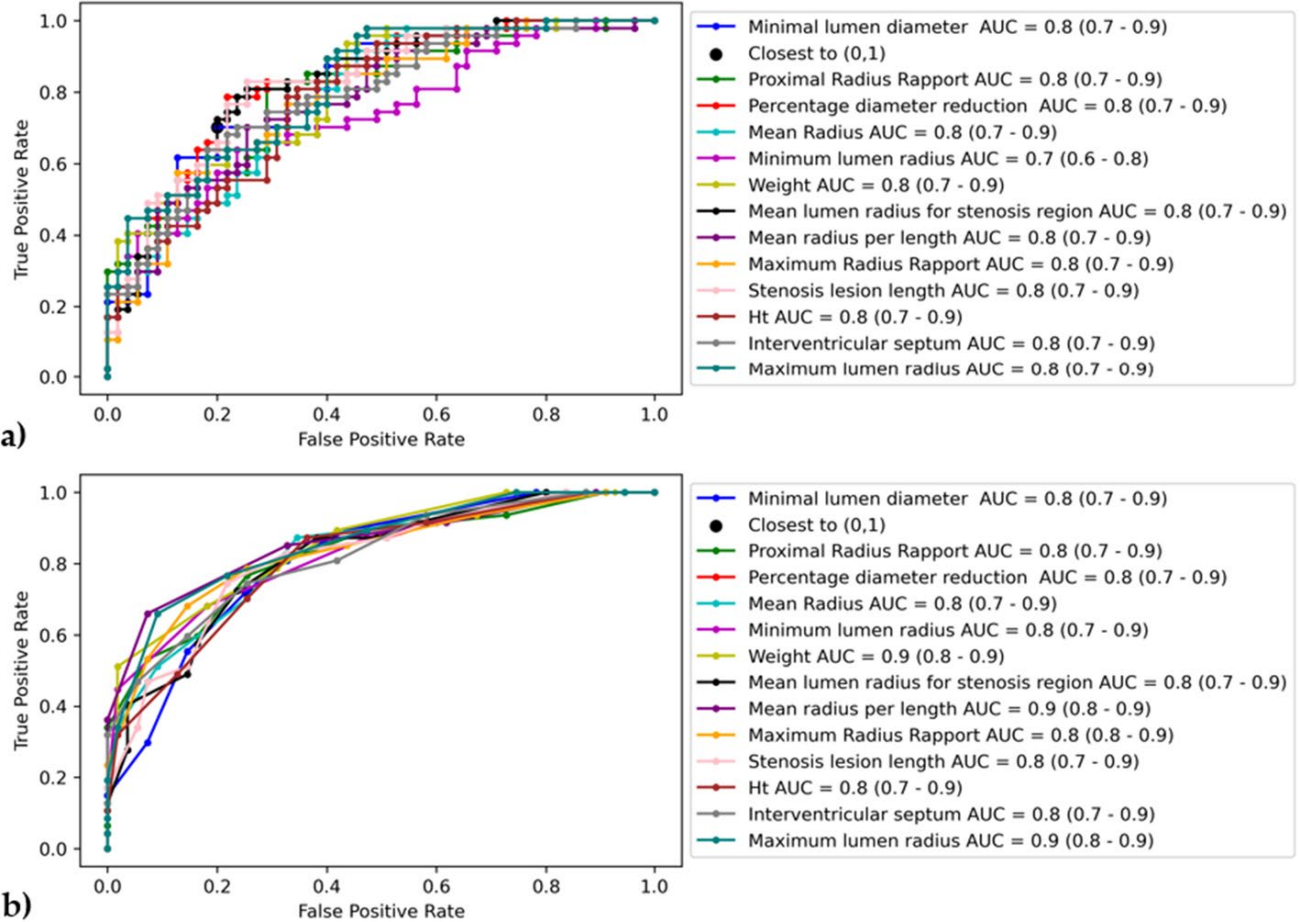
ROC curve with the optimal cutoff value along with the AUC score for 13 features ensemble model: **a** class probability and **b** prediction output

The best accuracy was obtained for 5 features (minimal lumen diameter, proximal radius rapport, percentage diameter reduction, mean radius, minimum lumen radius) ensemble model with 81.37% (Table 6). The ROC analysis for both crisp labels and class probabilities are further displayed in Fig. 4.

**Table 6:** The evaluation metrics computed for the best ensemble model with a confidence interval of 95%

Evaluation metrics	Values
Sensitivity (%)	80.85 (67.46,89.58)
Specificity (%)	81.82 (69.67,89.81)
PPV (%)	79.17 (65.74,88.27)
NPV (%)	83.33 (71.26,90.98)

For a narrower gray zone of 0.75 ≤ FFR ≤ 0.83 [23, 24] the model has an accuracy of only 71.43%. Outside this interval, the model performed significantly better (Table 7).

**Table 7:** Diagnostic performances depending on FFR value

	FFR < 0.75 or FFR > 0.83	0.75 ≤ FFR ≤ 0.83
Accuracy (%)	85.14	71.43

The best ensembled model is further analyzed regarding its dependencies with other clinical parameters, such as sex, vessel type, if the vessel is proximal LAD, if the patient is hypertensive or if he has diabetes (Table 8).

**Table 8:** Diagnostic performances for different clinical parameters

	Vessel Type			Proximal LAD		Sex		Hypertensive		Diabetes	
	LAD	LCX	RCA	Yes	No	F	M	Yes	No	Yes	No
Accuracy (%)	78.95	85	84	87.1	78.87	82.35	76.47	81.33	81.48	81.43	81.25

The LAD vessels are harder to diagnose due to an accuracy of 78.95%, as well as, if they are not proximal. Additionally, the patient’s sex is a factor of discordances, quantified by a difference in accuracy of 6% between women and man. The presence of diabetes or hypertension give similar performances and they are not discordance factors.

A multivariate model was tested for the top 5 to 13 features and the best accuracy was obtained for random forest classifier on the top 13 features with an accuracy of 71.56% which is lower than in the aggregation approach.

## Discussion

### Machine learning in stenosis classification

The paper proposed a new ML model as a potential tool for deciding the lesion significance based on the patient clinical characteristics and features extracted from the radii profile achieved from the intracoronary OCT. In the study, it was assumed that this medical imaging technique will provide better quality data in the assessment of the radii profiles.

The results sustain the hypothesis that the most important features in the ensemble model are extracted from OCT imaging; hence, the best model uses the top 5 features, all related to the radii profile: the minimal lumen diameter, proximal radius rapport, percentage diameter reduction, mean radius, and minimum lumen radius.

The criterion for performance evaluation was considered the models’ accuracy due to balanced classes of the dataset. Nevertheless, the confidence intervals of the other ensemble models’ performances overlap, and the differences are not statistically significant.

There are no similar methods reported in the scientific literature, but a comparison is made with other papers related to the virtual FFR.

ML-based techniques were employed in [11] for 125 patients considering intracoronary OCT and FFR. The partition ratio was 5:1 for splitting the training and testing datasets. A random forest model was used, with the six most important features as inputs: MLA, percentage of the stenotic area, lesion length, proximal lumen area, preprocedural platelet count, and hypertension. The sensitivity, specificity, positive predictive value, negative predictive value, and accuracy of the OCT-based machine learning-FFR for the testing group were 100%, 92.9%, 87.5%, 100%, and 95.2%, respectively.

Another attempt considered in [14] by the same authors identified 36 features, including 16 clinical lesion characteristics and 20 OCT extracted features. The classification performances were assessed using sensitivity, specificity, positive predictive value, negative predictive value, and accuracy as 85.7%, 100%, 100%, 77.8%, and 90.5%, respectively.

Even if the accuracies of these models are higher, it must be noted that the splitting ratio of 5:1 for training versus testing inherit a risk of generalization lack. In the present study, with almost the same number of cases, the leave group out approach was chosen for preventing this issue.

All these papers don’t mention if their private dataset have FFR values inside the gray zone.

A study aimed to evaluate the OFR diagnostic performance [17] in comparison with the angiography-based QFR, having the wire-based FFR as a gold standard for 212 vessels from 181 patients with an average FFR of 0.82 ± 0.10 from which 40.1% of vessels had an FFR ≤ 0.80. The diagnostic accuracy, sensitivity, and specificity of OFR to identify FFR ≤ 0.80 were 92%, 86% and 95%, respectively. The computational burden of this algorithm is one of the most important issues.

The study [18] tends to be more relevant in our comparison, used an angiography-based ML algorithm for classifying the lesions significance using the FFR cutoff value with an overall accuracy of 82% and AUC of 0.87 for 1501 patients with 1501 intermediate lesions.

In the scientific paper [25], a deep learning (DL) approach was used for estimating the FFR based on the CFD simulations applied to synthetically generated coronary anatomy with an accuracy of 83.2%.

The best comparison with the current study that used the same OCT dataset, is reported in [26], which used DL algorithms applied on raw radii profiles extracted from the images. Few-shot learning reached the highest accuracy of 77.5%. The present model outperformed the DL-based model due to an enhanced dataset that included the clinical characteristics of the patient.

An independent comparison study [27] was done for 5 software/methods of angio-FFR estimation on a large dataset. The ROC analysis was performed for each method for detecting the FFR ≤ 0.8 and the AUC values were between 0.73 and 0.75, which are lower than the previously diagnostic performances reported in their validation studies.

Although the model accuracy of 81.37% is slightly lower than ones from the other studies which reported accuracies ranging from 82% to 95.2%, it is essential to consider that our cohort consisted of nearly 50% stenoses in the gray zone, with an FFR of approximately 0.8, unlike the others with a smaller representation in this region. Still, for cases with FFR outside the gray zone, the model reached an accuracy of 85.14%.

Our method claims a better computational time, due to the fact it does not need the 3D modeling reconstruction, CFD simulations or deep learning implementation, making it suitable for a further development in the clinical practice.

Ensemble learning reduces the generalization error and improves the overall performance of the individual model accuracy from an average of 67.35% to 81.37%. Its inputs were the crisp labels (significant lesion or not significant) for all features using the mean ROC cutoff value approach.

The ensembled model boosted the model performances in comparison to the multivariate model from 71.56% to 81.37%.

From the current study, the following interpretations can be concluded:

- measurements related to the stenosis region are the dominant features due to their strong correlation with FFR,
- weight is an important physiological characteristic of the patient.
- features related to the lesion region have an important impact on the classification output,
- AUC score for the crisp labels outperforms the class probability approach.

### Clinical implications

To evaluate the clinical impact, the study focused only on non-culprit lesions of patients with ACS and multivessel disease. There were no other restrictions or regulations regarding the data acquisition which offer a broader perspective of the clinical applications.

The explainability goal was targeted for a better understanding and acceptance as decision support in daily clinical practice.

The accuracy for a narrower gray zone of 0.75 ≤ FFR ≤ 0.83 for clinical decision making of percutaneous coronary intervention (PCI) reached 71.43% and it can suggest that the invasive FFR measurement is mandatory for the critical region, whereas, for the cases of outside this interval, the model performance improves substantially to an accuracy of more than 85%.

Therefore, the model offers good performances outside the gray zone that can guide the decision making for choosing the proper treatment that may include the immediate revascularization or taking medications.

For the cases inside the interval, the algorithm suggests the need for getting extra information about the lesion type by performing the invasive FFR measurement. Nevertheless, additional medical information may also include the evaluation of the coronary flow reserve which imply the coronary blood flow velocity measuring. This can be done both invasively using an ultrasound transducer-based catheter or non-invasively, using positron emission tomography (PET).

The importance of the study is given by the fact that it is not possible to rely only on 2D invasive coronary angiography and FFR measurement for treating non-culprit lesions. FFR measurement is subject to artefacts which will yield inaccurate results and it’s producing a hemodynamic disturbance due to the hyperemia inducing state [28]. Moreover, having a model that could simplify the amount of information coming from all invasive assessments (OCT or FFR) could be a real help for interventionists who must take important and instant decisions on treating patients in cardiac catheterization labs.

### Clinical implementation

To implement the model in a clinical setting, we envisage the followings: patients are included only after signing an informed consent form; a set of initial inclusion and exclusion criteria are also checked, and if they are met, the XA and OCT exams are performed; data is then extracted and annotated using the dedicated tool.

The XA and OCT data are processed using a cloud-based or on-premise application and the prediction model will run using an artificial intelligence service. Finally, the prediction outputs are interpreted by the clinical expert, who then takes the final diagnosis and treatment decision.

### Limitations

The study was limited by a single-center acquisition and there was no other dataset similar to be found in public databases, which constrained the current study to only 80 patients. Finally, to generalize the proposed model, further validation is necessary in a large multicenter cohort of subjects of different races.

The ground truth that is built based on a fixed FFR cutoff value, forced the inclusion of confidence intervals to make them more clinically relevant.

The lumen geometry extracted from OCT imaging may be influenced by the errors in the acquisition process, by the contour detection and its corrections performed in the built-in software.

### Contributions and further perspectives

The study contributions include new features extraction from intracoronary OCT (Table 9) capable of quantifying stenosis lesion severity and building the ensemble model obtained using the ML approach for classifying the significance of coronary lesions.

**Table 9:** Features computed directly from the OCT images

No	Name	Formula
1	Mean lumen radius	*r*_mean_ = average of radii along the vessel segment
2	Minimum lumen radius	*r*_min_
3	Maximum lumen radius	*r*_max_
4	Mean lumen radius for stenosis region	Average of radii along the stenosis region
5	Mean radius per length	*r*_mean_/vessel segment length
6	Maximum radius rapport	(*r*_max_ − *r*_min_)/*r*_max_
7	Stenosis lesion length	lengthS
8	Percentage diameter reduction	$100*\left(1-\frac{2*{r}_{{\text{mean}}}}{{r}_{{\text{proximal}}}+{r}_{{\text{distal}}}}\right)$
9	Proximal radius rapport	${{\left( {r_{r_{{\text{proximal}}} } - r_{{\text{distal}}} } \right)} / {r_{r_{{\text{proximal}}} } }}$
10	Radius rapport	${{\left( {\left( {r_{{\text{proximal}}} + r_{{\text{distal}}} } \right)/2 - r_{{\text{min}}} } \right)} / {\left( {\left( {r_{{\text{proximal}}} + r_{{\text{distal}}} } \right)/2} \right)}}$
11	Proximal area	$\pi {{r}_{\mathrm{proximal }}}^{2}$
12	Distal area	$\pi {{r}_{\mathrm{distal }}}^{2}$

A further perspective includes the extension of the dataset at least with 100 new patients for testing other ML strategies for preserving the explainability goal, which creates a classification mechanism that is interpretable and can be reproductible by humans.

The inclusion of other clinical biomarkers and angiography results can contribute to designing a holistic model for clinical decision making of vascularization.

## Conclusions

OCT-based machine learning lesion classification can be used to acquire morphological and functional information into a single procedure, suggesting that it may enhance the treatments of coronary artery stenoses.

The paper introduces a single-center study for evaluating the importance of intermediate coronary lesions that may lead to ischemia in the major coronary arteries. The assessment primarily involves extracting features from both OCT images and patient characteristics.

## Methods

### Data acquisition

The dataset was collected from the Clinical Emergency Hospital, Bucharest, Romania, and it was conducted in compliance with the Declaration of Helsinki for investigation in human beings.

The study protocol was approved by the local ethics committee of the hospital, after all patients gave their written informed consent before enrollment.

The dataset comprises 80 patients with 102 lesions with stable coronary artery disease or acute coronary syndrome (ACS) and multivessel disease. Only non-culprit lesions were considered in ACS patients. Culprit lesions were examined and treated during hospitalization, whereas non-culprit lesions were evaluated based on XA, OCT and invasive FFR during a second hospitalization, generally after two weeks.

OCT imaging was performed using Optis (St. Jude Medical/Abbott, St. Paul, MN, USA) and Dragon Fly catheters. The fiber probe was pulled back at a constant speed, and cross-sectional images were acquired with 5 frames/mm during manual contrast injection. The maximum vessel length that can be evaluated during one pullback is 75 mm.

FFR measurement was performed using a Quantum system (St. Jude Medical/Abbott, Minneapolis, MN, USA). The measurement was performed after the administration of adenosine, either intravenously at a constant rate of 140 μg/kg/min or as an intracoronary bolus (50–100 μg for the right and 100–400 μg for the left coronary artery) [29].

The clinical protocol states that after recording FFR, the pressure wire is pulled back with the sensor at the tip of the guiding catheter to measure the pressure drift. If these values differed by more than ± 3 mmHg, pressures must be re-equalized, and the measurements are repeated.

Echocardiography was performed for all patients, and the relevant clinical measurements were given by the physician: ejection fraction (echo EF), diastolic pattern, and interventricular septum size. From the blood tests, hemoglobin (Hb) and hematocrit (Ht) levels are used in the study.

### Data processing

The OCT images were anonymized and exported in Digital Imaging and Communications in Medicine (DICOM) format [30] with a spatial resolution of 704 × 704 pixels.

The images were analyzed by the clinical team, and the inner vessel contour was automatically traced in the OCT console. Another verification was done on acquired data and some slices with improper contours were eliminated or they had been corrected in a built-in software.

The interventional cardiologist annotated the dataset as follows:

- OCT frames related to the proximal and distal region of the vessel segment,
- OCT frames related to the proximal and distal region of the lesion,
- tracing the lumen border from the medical imaging device under medical imaging expert guidance for computing the vessel’s radii and the estimation of MLD.

**Fig. 5:**
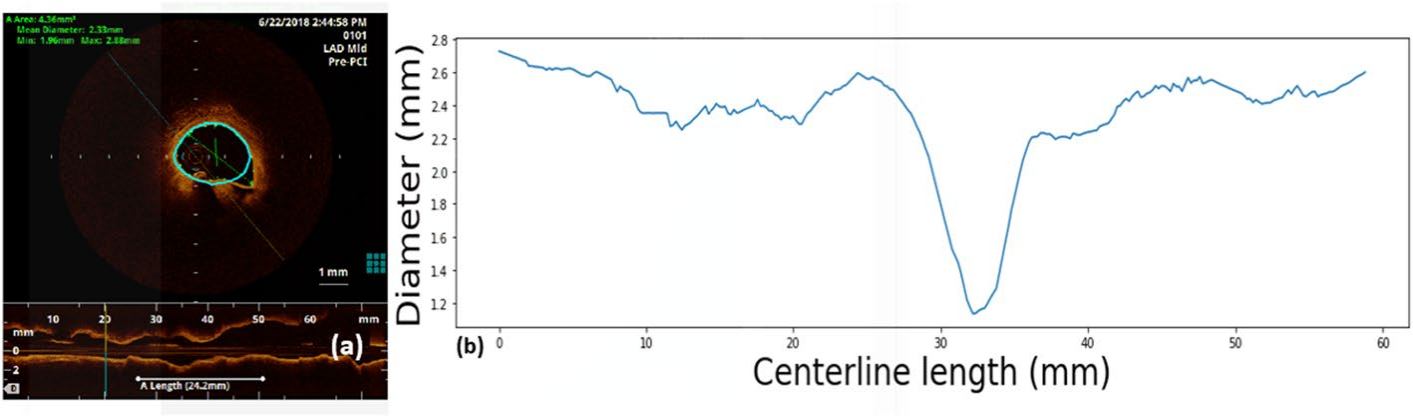
**a** OCT slice with lumen border tracing, the corrected contour is represented in light blue by the built-in software and the contour provided by the machine in orange, **b** Unidimensional signal with the evolution of the diameters extracted from the OCT slices along the vessel length

Figure 5a is a sample of OCT slice with the coronary artery lumen tracing after contour correction, which is used to build the unidimensional signal of the diameters’ evolution along the vessel length (Fig. 5b).

The 12 features mentioned in Table 9 are computed directly from that unidimensional signal, considering the proximal and distal radii of the lesion (r_proximal and r_distal respectively) given by the medical expert.

The difference between MLD and minimum lumen radius is that the first one is estimated by the physician and the last one is computed directly from the evolution of the diameters along the vessel length (Fig. 5b). This signal is subject to changes due to the OCT imaging preprocessing done in the built-in software, where the contours are manually adjusted when it was needed.

Thirteen clinical characteristics of the patients were used as features in the algorithm: sex, age, height (cm), weight (kg), smoking (0 no; 1 past; 2 present), AHT, dyslipidemia, diabetes, ejection fraction, diastolic pattern, interventricular septum size, hemoglobin, and hematocrit levels.

The ground truth is related to FFR measurement, which has been corrected previously considering the pressure drift (D), which represents the arithmetic difference between aortic pressure (drift_Pa) and distal pressure (drift_Pd). The drift is done at the end of the measurements to check the pressure curves if they are superimposed [31].

Even if drift values of 1–2 mmHg are generally accepted as insignificant in medical practice [32], a drift of 2–3 mmHg was considered relevant in the study, improving the models’ accuracy. Hence, a correction of the measured distal pressure (corrected_Pd) was introduced considering the drift measurement:

1 $$ {\text{Corrected}}\_{\text{Pd}} = {\text{Pd}} + D $$

where Pd is the distal pressure measured at the beginning of the FFR investigation.

The updated value of FFR (${\text{corrected}}\_{\text{FFR}}$), which is measured at maximal hyperemia, will be computed as in the medical literature [21]:

2 $$ {\text{Corrected}}\_{\text{FFR}} = \frac{{{\text{corrected}}\_{\text{Pd}}}}{{{\text{Pa}}}} $$

where Pa is the aortic pressure measured at the beginning of the invasive FFR investigation.

Only 15 lesions out of 102 were adjusted according to the above-mentioned formula (2). Twelve lesions had a drift variation of 2 mmHg, and only 3 lesions had a drift variation of 3 mmHg. The correction value influenced the ground truth for two lesions by underestimating the initial value of 0.8 and setting the case into the positive class.

The medical data were extracted both from the clinical records and from the medical images as following:

- OCT imaging and invasive FFR for each one of three coronary arteries (RCA, LCX or LAD) were performed in PCI during the angiography procedure,
- The FFR value was corrected using Eq. (2),
- A database was constructed in which the cardiologist recorded the start and end of the OCT frames to delimitate the vessel segment, the start and end of the OCT slices corresponding to the lesion,
- Patient characteristics were extracted from the clinical records: physiological, anatomical, and behavioral features correlated to coronary artery disease.

### The algorithm overview

The ground truth is computed based on the corrected value of FFR into two classes, if the coronary lesions are hemodynamically significant or not:

3 $$ y{ } = \left\{ {\begin{array}{*{20}c} {0,} & {{\text{Corrected}}\_{\text{FFR }} > 0.8} \\ {1,} & {{\text{Corrected}}\_{\text{FFR }} \le 0.8} \\ \end{array} } \right. $$

The feature matrix was first normalized using L1 norm or Manhattan distance which computes the sum of the magnitudes of the features.

Twelve base learners were tested for 10 different seeds, such as support vector machine (SVM) with different kernels, decision tree, random forest with different number of trees, k-nearest neighbors, AdaBoost, logistic regression or naive Bayes [33–35]. The usage of the same random seed per model computation for each type of classifiers, can guarantee a precise, reproducible, and uniform evaluation of each algorithm.

The leave-group out method [36] was applied at the patient level, hence 80 training/testing folds for each model computation, as follows: for each fold, a threshold value was set to balance sensitivity and specificity on the respective training set. Finally, the chosen threshold is applied to classify the test sample(s).

To evaluate the results, diagnostic statistics [37] were computed for all approaches: accuracy, sensitivity, specificity, negative predictive value (NPV), and positive predictive value (PPV).

The accuracy is computed as the ratio between the correct predictions and all samples’ predictions, as revealed in relation (4):

4 $$ {\text{Acc}} = \frac{{{\text{TP}} + {\text{TN}}}}{{{\text{TP}} + {\text{TN}} + {\text{FP}} + {\text{FN}}}} $$

where TP is the true positive, TN is the true negative, FP is the false positive and FN is the false negative.

The area under the curve by receiver-operating characteristic (ROC) analysis [38] was used on mean probabilities of the positive or negative class (significant or nonsignificant lesion) and on mean prediction output for all models. Instead of the popular Youden index, the closest to (0,1) criteria [39] gave better results in identifying the best cutoff value for the given inputs. Each optimal threshold and its average are further used in constructing the final model.

Ensemble learning groups different models for solving difficult problems to improve the overall performance accuracy and reduce the variance at the cost of increasing the bias [40, 41].

For feature analysis and dependencies, the correlation matrix was computed based on Pearson correlation [42]. Percentage diameter reduction and radius rapport revealed a perfect correlation, hence, the last one was eliminated from the feature map.

**Fig. 6:**
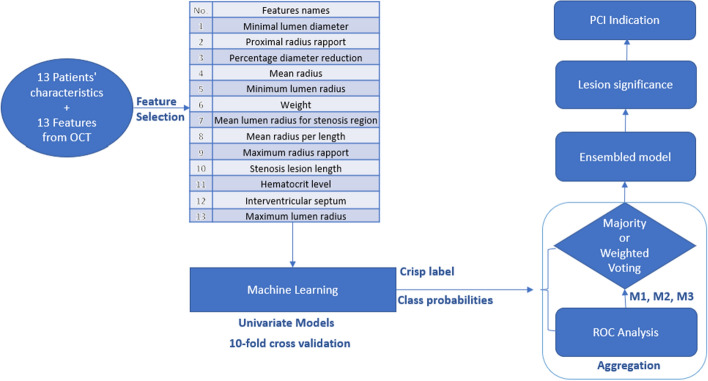
Algorithm overview of the ensembled model

The following experiments were conducted considering an ensemble model made of 13 univariate models obtained for each feature individually illustrated in Fig. 6.

Based on models’ performance, eleven features were extracted from the intracoronary OCT frames: MLD, proximal radius rapport, percentage diameter reduction, mean radius, minimum lumen radius, mean lumen radius for stenosis region, mean radius per length, maximum radius rapport, stenosis lesion length, and maximum lumen radius. In addition to that, the weight, the hematocrit level, and the interventricular septum are added into the final feature map.

Considering the univariate models of the top 13 most relevant features obtained after tenfold cross validation, a multi-feature voting is used for building the ensemble model. ROC analysis was applied, and the ensemble predictions were computed by averaging the output prediction of all 10 models.

The output of the class label (Oi) can take different types of values according to the information provided by the classifiers C_i_:

5 $$ O_i \in \left\{ {\begin{array}{*{20}c} {\left\{ {0,1} \right\},} & {{\text{crisp}}\;{\text{label}}} \\ {\left[ {0,1} \right],} & {{\text{class}}\;{\text{probability}}} \\ \end{array} } \right. $$

In the scientific literature [41] it is suggested that the class probabilities estimated by most classifiers have, in general, lower performance, except for the situation of a careful calibration.

Considering the crisp label and the calibrated class probability as inputs for the ROC analysis, the final outputs of each model are used in a voting system to obtain the three different aggregation strategies:

- M1—the mean ROC cutoff value of the class probabilities of all univariate models is the threshold of the final predictions that are further used as inputs into the voting system.
- M2—the cutoff values after the ROC analysis of each corresponding feature model are the thresholds for the resulting models that are further used as inputs into the voting system.
- M3—the optimal cutoff value obtained after the ROC analysis of the crisp labels of each univariate model is the threshold for the final models that are further used as inputs into the ensemble model.

For comparison, a simple majority vote and a weighted voting [43] are implemented in the classification system having as input the mean of the models’ outputs after performing the ROC analysis.

In the simple majority vote approach, every classification model votes for one class label, and the final output class label is the one that receives more than half of the votes.

As implementation, the ensemble model’s output is given by the combination of each one of the 13 univariate models that can vote. A 0.5 threshold is applied to their mean for computing the final prediction output.

Weighted voting assumes that the individual classifiers have unequal performance and it will give more voting power to the stronger classifiers. The weights should be proportional to the performance of the individual learners.

The optimal weights (w_i_) assigned to the classifier (C_i_) are computed from [43] with the following formula (6):

6 $$ w_i = \log \frac{{{\text{acc}}_i }}{{1 - {\text{acc}}_i }} $$

where acc_i_ denotes the accuracy of the classifier C_i_ and $i=\stackrel{-}{\mathrm{1,13}}$ features.

Finally, the mean of each output (O_i_) is multiplied by the corresponding weight and compared to threshold value of 0.5 to compute the ensembled model output.

The implementation of the algorithms was performed using the scikit-learn Python library [44].

## Data Availability

The data that support the findings of this study are available from the local ethics committee of the Clinical Emergency Hospital, Bucharest, but restrictions apply to the availability of these data, which were used under license for the current study and are not publicly available. Data are, however, available from the corresponding author I.-A.T. upon reasonable request and with permission of the local ethics committee of the Clinical Emergency Hospital, Bucharest.

## Abbreviations

ACS: Acute coronary syndrome
AHT: Arterial hypertension
AUC: Area under the curve
CAD: Coronary artery disease
CFD: Computational fluid dynamics
DICOM: Digital Imaging and Communications in Medicine
DL: Deep learning
EF: Ejection fraction
FFR: Fractional flow reserve
FN: False negative
FP: False positive
Hb: Hemoglobin level
Ht: Hematocrit level
IVS: Interventricular septum
IVUS: Intravascular ultrasound
LAD: Left Anterior Descending artery
LCx: Left circumflex artery
ML: Machine Learning
MLA: Minimal lumen area
MLD: Minimal lumen diameter
NPV: Negative predictive value
OCT: Optical coherence tomography
OFR: OCT-based optical flow ratio
PCI: Percutaneous coronary intervention
PET: Positron emission tomography
Poly SVM: Support vector machine with polynomial kernel
PPV: Positive predictive value
QCA: Quantitative coronary angiography
QFR: Angiography-based quantitative flow ratio
M1: Class probabilities for all features using the mean ROC cutoff values
M2: Class probabilities using the corresponding ROC cutoff for each feature
M3: Predicted output for all features using the mean optimal ROC cutoff value
RCA: Right coronary artery
RF: Random forest
ROC: Receiver-operating characteristic
SVM: Support vector machine
TN: True negative
TP: True positive
XA: X-ray Coronary Angiography
